# Identification, Characterization, and X-ray Crystallographic Analysis of a Novel Type of Mannose-Specific Lectin CGL1 from the Pacific Oyster *Crassostrea gigas*

**DOI:** 10.1038/srep29135

**Published:** 2016-07-05

**Authors:** Hideaki Unno, Kazuki Matsuyama, Yoshiteru Tsuji, Shuichiro Goda, Keiko Hiemori, Hiroaki Tateno, Jun Hirabayashi, Tomomitsu Hatakeyama

**Affiliations:** 1 Graduate School of Engineering, Nagasaki University, 1-14 Bunkyo-machi, Nagasaki 852-8521, Japan; 2 Research Center for Medical Glycosciences, National Institute of Advanced Industrial Science and Technology, Tsukuba 305-8568, Japan

## Abstract

A novel mannose-specific lectin, named CGL1 (15.5 kDa), was isolated from the oyster *Crassostrea gigas*. Characterization of CGL1 involved isothermal titration calorimetry (ITC), glycoconjugate microarray, and frontal affinity chromatography (FAC). This analysis revealed that CGL1 has strict specificity for the mannose monomer and for high mannose-type N-glycans (HMTGs). Primary structure of CGL1 did not show any homology with known lectins but did show homology with proteins of the natterin family. Crystal structure of the CGL1 revealed a unique homodimer in which each protomer was composed of 2 domains related by a pseudo two-fold axis. Complex structures of CGL1 with mannose molecules showed that residues have 8 hydrogen bond interactions with O1, O2, O3, O4, and O5 hydroxyl groups of mannose. The complex interactions that are not observed with other mannose-binding lectins revealed the structural basis for the strict specificity for mannose. These characteristics of CGL1 may be helpful as a research tool and for clinical applications.

As with many invertebrates, resistance against infectious diseases in shellfish is provided by an innate immune system. In the innate immune system of bivalves such as oysters, clams and mussels, pattern recognition receptors are commonly associated with host defense responses against infection. These pattern recognition receptors include lectins, peptidoglycan-recognition proteins, thioester bearing proteins, lipopolysaccharide and β-glucan binding proteins, and fibrinogen-related proteins^1^. Remarkably, lectins are thought to play a crucial role in the innate immune system through specific binding to polysaccharide-coated pathogenic bacteria. Several lectins have been identified in bivalves and they most frequently belong to the C-type lectin and galectin families, two of the major animal lectin groups with conserved folds^2^,^3^. Lectins are ubiquitous in living organisms (from microorganisms and viruses to humans). Until now, 48 lectin families have been reported in scientific papers that have focused on a variety of tertiary structures and recognition mechanisms for specific carbohydrates^4^. Some of the lectin families are outspread in distant species and include lectins with a variety of specificities for carbohydrates. Lectins capable of binding with mannose have been confirmed in a number of lectin families, where most of these do not have strict specificity for mannose alone. For example, mannose-binding lectins from rat serum (MBP-C) and rat liver (MBP-A) have a broad carbohydrate specificity, including that for mannose, N-acetylglucosamine, and fucose^5^,^6^. In addition, it was also reported that MBP-C and concanavalin A interacts with the trimannosyl core of the complex N-linked oligosaccharides^7^,^8^. BanLec from banana, GNA from bulbs, and BC2L-A from bacteria show binding specificity for mannose monomers and high mannose-type N-glycans HMTGs^9^,^10^,^11^. Some lectins, including actinohibin^12^ from actinomycetes and Cyanovirin-N^13^ from cyanobacteria, have demonstrated binding specificity only for HMTGs. While there are various folding patterns of these lectins and binding regions on the HMTGs, these lectins also exhibit anti-HIV activity by binding to the HMTGs on gp120, an envelope protein of HIV^14^. Therefore, lectins with binding specificities for HMTGs can be used as potential microbicides in preventing HIV transmission.

In the present study, we purified a mannose-binding lectin named CGL1 from a pacific oyster *Crassostrea gigas*, characterized its sugar-binding specificity, and determined primary and crystal structures. Characterization of CGL1 revealed a strict specificity for the mannose monomer and HMTGs. Primary and tertiary structures do not have any similarity with those of the known lectins but do have similarity with the primary structures of the proteins in the natterin family whose function appears to involve toxicity^15^,^16^. Analysis of complex structures of CGL1 with mannose revealed a mechanism for its binding and a structural basis for the specificity.

## Results and Discussion

### Purification of CGL1 from *C. gigas*

**Figure 1:**
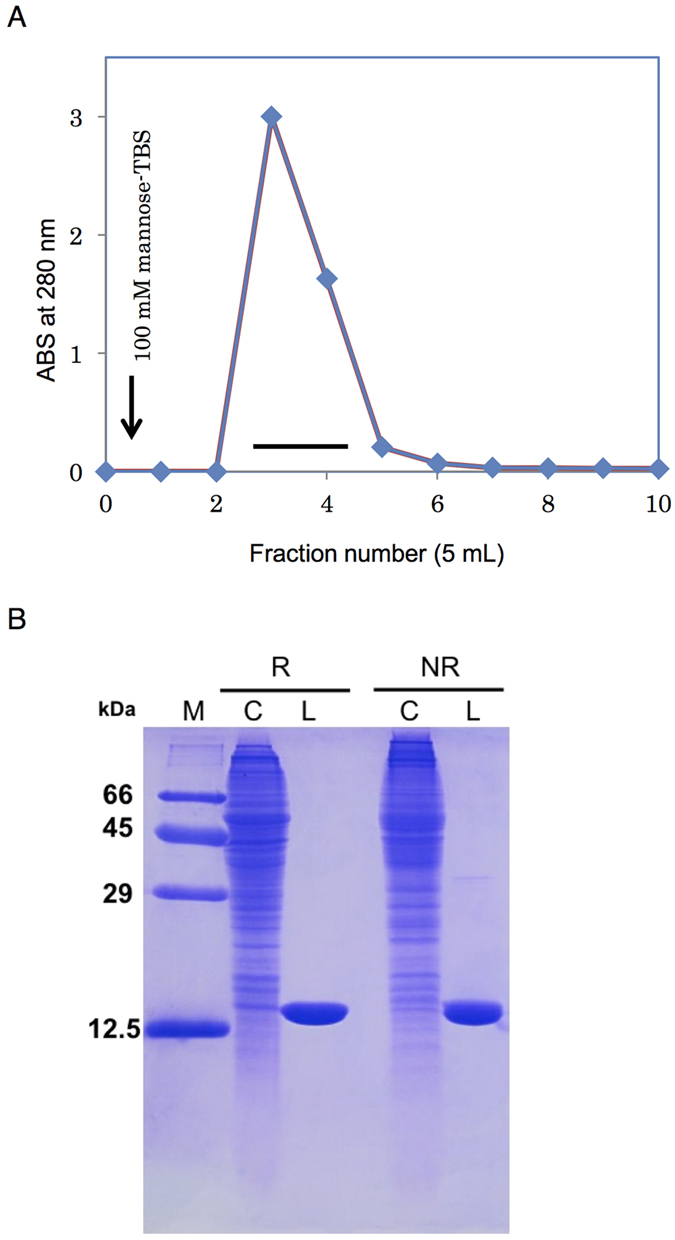
Purification of CGL1. (**A**) TBS extract from the Atlantic oyster (*Crassostrea gigas*) was applied to a mannose-conjugated cellulose column (1.4 × 3.5 cm) equilibrated with TBS. CGL1 that was bound to the column was eluted with TBS containing 100 mM mannose (*arrow*). ABS: absorbance. (**B**) an SDS-PAGE pattern under reducing (*R*) and nonreducing (*NR*) conditions. Numbers on the left indicate the molecular weights of marker proteins as follows: BSA (66 kDa), ovalbumin (45 kDa), carbonic anhydrase (29 kDa), and cytochrome C (12.5 kDa). M, molecular marker; C, crude extract; and L, lectin.

During affinity chromatography of the crude supernatant applied to the mannose-Cellulofine column, a single peak appeared during elution with TBS containing 100 mM mannose (Fig. 1A). SDS-PAGE showed this lectin as a single polypeptide with molecular weight of 14 kDa under reducing and nonreducing conditions (Fig. 1B). The protein was further purified by gel filtration and dialyzed. The processing of 500 g of *C. gigas* tissue yielded 10 mg of lectin. The 14-kDa lectin purified from *C. gigas* was therefore named CGL1 (***C**rassostrea **G**igas* **L**ectin **1**).

### Sugar-binding specificity and affinity of CGL1

The purified CGL1 (native CGL1; nCGL1) did not show any significant hemagglutinating activity with rabbit, horse, bovine, chicken, or sheep erythrocytes (data not shown). Therefore, for characterization of interactions between CGL1 and saccharides, stoichiometry and affinity were measured using ITC (Table 1, Supplementary Fig. S1). Mannose, αMeMan, and mannobiose (Manα1-2Man) have affinity characterized by the association constants 2.00 ± 0.46 × 10^3^ M^−1^, 1.49 ± 0.50 × 10^3^ M^−1^, and 6.00 ± 0.99 × 10^3^ M^−1^, respectively. No affinity was detectable for the other monosaccharides (galactose, glucose, rhamnose, GlcNAc, GalNAc, galactosamine, NeuNAc fucose, αMeGal, and βMeGal). The hemagglutination assay of CGL1 did not show any significant activity toward erythrocytes of several animal species, although oligosaccharides on the surface of erythrocytes contain mannose in the core structure^17^. This observation indicates that CGL1 should bind only to the terminal mannose in oligosaccharides. Exploration of the specificity and recognition of structures in the oligosaccharides by CGL1 are described in a later section.

### Glycoconjugate microarray

**Figure 2:**
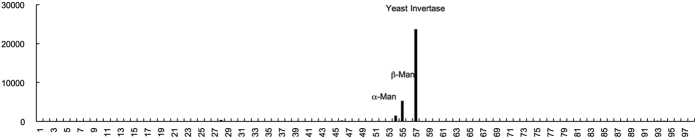
Glycoconjugate microarray analysis for sugar-binding specificity of CGL1. Cy3-labeled CGL1 was used to probe the microarray. The net intensity value of each spot represents the signal intensity minus the background value (Cy3-labeled BSA). The oligosaccharides are indicated by numbers that correspond to those shown in Supplementary Table S1.

In order to investigate the binding specificity of CGL1 against a wide range of carbohydrates, we performed glycoconjugate microarray analysis (Fig. 2 and Supplementary Table S1). Using a series of complex oligosaccharides, this analysis revealed significant binding for only 3 types of carbohydrates, α-mannoside, β-mannoside, and HMTGs (from yeast invertase). The strongest signal was especially observed for yeast invertase (entry 57), followed by β-mannoside (entry 55) and α-mannoside (entry 54). The carbohydrate chains of yeast invertase were composed of Man_8–14_GlcNAc, which is an N-glycan containing a trimannosyl core, and terminal mannose residues^18^. In contrast, complex-type N-glycan containing the trimannosyl core and different terminal sugars^19^,^20^, did not show any significant binding (entry 28, 46, 51). These results indicate that CGL1 binds very specifically to HMTG and the mannose monomer, For a more detailed analysis of the binding profile and for the determination of its association constant, we performed FAC analysis with oligosaccharides, including a series of HMTGs.

### Oligosaccharide specificity of CGL1 analyzed by FAC

**Figure 3:**
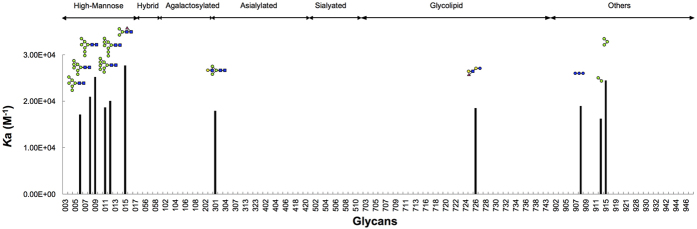
Affinities of PA and pNP glycans for CGL1 as assessed by FAC. The analysis was performed using 125 oligosaccharides. The affinities of the oligosaccharides are represented as association constant (*K*a) values on the left scale. The oligosaccharides are indicated by numbers that correspond to those shown in Supplementary Fig. S2.

Figure 3 shows the carbohydrate binding specificities of CGL1 for various oligosaccharides analyzed by FAC. In this analysis, fluorescence-labeled PA or pNP oligosaccharides (Supplementary Fig. S2) were applied to a column containing the immobilized lectins, and the retardation of their elution (*V*–*V*_0_) caused by interactions between their oligosaccharides and the lectin was measured to determine their affinities. As shown in Fig. 3, CGL1 exhibited significant affinities for oligosaccharide numbers 006, 008, 009, 011, 012, 015, 301, 726, 908, 914, and 915. These oligosaccharides, except numbers 726 and 908, contain terminal mannosides in their complex carbohydrates. On the other hand, oligosaccharides containing a trimanniside core structure with other sugars on its terminal residues, i. e., the oligosaccharide numbers from 103 to 203 and from 304 to 510, does not show any affinity. This indicates that CGL1 does not bind to the trimannosyl core structure, but to the terminal mannose residues in the oligosaccharides. This is consistent with the results obtained from analysis of the glycoconjugate microarray described above. FAC analysis with a series of HMTGs showed that CGL1 could not bind to all of the HMTGs, but with some of the HMTGs, i. e., the oligosaccharides numbers 006, 008, 009, 011, 012, 015. This indicates that CGL1 would not bind to all of the terminal mannose residues, but to particular structures of these in HMTGs. In terms of these particular structures, none of the definite patterns in the terminal structures of these HMTGs could be confirmed, yet we found that these HMTGs tend to include α1–2 bonds at terminal mannoside residues. In addition to the binding affinities for oligosaccharides containing the terminal mannoside, CGL1 also exhibited significant affinities for oligosaccharide numbers 726 and 908, which do not contain any mannose residues. This suggests that CGL1 can also bind to some specific oligosaccharides without mannoside residues, which might be other potential targets for CGL1.

Most of the mannose-specific lectins do not have a strict specificity for mannose alone. On the other hand, there are some lectins that have a binding specificity for HMTGs (HMTG-binding lectins). These lectins are able to recognize multiple mannosides in 1 HMTG at a time, while they fail to demonstrate detectable binding to mannose monomer because of its low affinity for monosaccharides^14^. In comparison with the known mannose-specific and HMTG-binding lectins, binding specificity of CGL1 considerably differ from that of these lectins. For elucidation of the structural basis for the unique specificity of CGL1, structural analyses including crystal structure of CGL1-mannose complex were carried out as described below.

Some of the reported HMTG-binding lectins demonstrate anti-HIV activity induced by its binding to HMTGs of gp120, an envelope glycoprotein of HIV^12^,^14^. HMTGs are rare in normal mammalian cells, thus, CGL1 would be a good candidate that can be further investigated as a potential and safe microbicide against viruses and microbes bearing HMTGs on its envelopes.

### Primary Structure of CGL1

**Figure 4:**
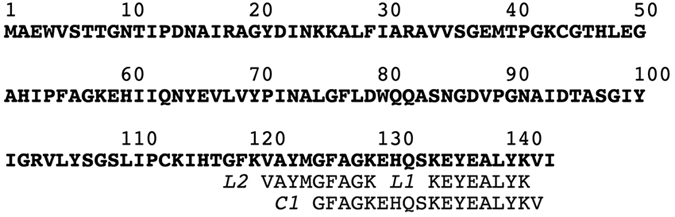
The amino acid sequence of CGL1. The proven sequence of CGL1. Sequences that were determined by Edman degradation of specific peptides (indicated by italic type) are shown by means of 1-letter code. The identified full length of the CGL1 sequence from the gene EKC36293.1 according to a BLAST search with a partial sequence from the peptides is shown in boldface.

Edman degradation of CGL1 did not yield any N-terminal amino acid, suggesting that this lectin had a blocked amino terminus. Therefore, to obtain information on the internal sequence, we cleaved the protein by an enzyme (lysyl endopeptidase) and a chemical agent (CNBr) to generate peptide fragments. Several peptides were obtained from a digest of CGL1 after separation by reverse-phase HPLC (Supplementary Fig. S3). Sequence analysis of the 3 peptides yielded 35 amino acid residues: L1, KEYEALYK; L2, VAYMGFAGK; and C1, GFAGKEHQSKEYEALYKV. These peptide sequences were used for a BLAST search against the *C. gigas* genome database^21^ to identify the gene and the full length of the amino acid sequence. The gene turned out to be EKC36293.1, which encodes an amino acid sequence consisting of 143 amino acid residues (Fig. 4), and its molecular mass was determined to be 15.5 kDa. The amino acid sequence of CGL1 did not share any homology with that of known lectins. Accordingly, CGL1 was assumed to be a novel lectin with strict specificity for mannose. On the other hand, the protein sequence of CGL1, which is named “natterin-3” (Entry: K1QRB6) in the Uniprot database^22^, shares a 26–29% identity with the N-terminal region of natterin proteins from the venom of the *Thalassophryne nattereri* fish (Supplementary Fig. S4), which was identified as a new protein family, natterin family, with kininogenase activity^15^. In addition, it was also reported that Toxin I and II from the skin secretion of the oriental catfish *Plotosus lineatus* belong to the natterin family and have lethal toxicity and edema-forming and nociceptive effects^16^. Nineteen kinds of protein sequences that were translated from the genomic information from a wide range of species (fishes, fungi, rotifers, and insects) share homology with natterin and are assigned to be members of the natterin family in the database. Protein lengths in the natterin family are subdivided into 3 groups: short (104–156 amino acid residues) including CGL1 (143 amino acid residues), middle (312–387 amino acid residues), and long (576 amino acid residues); the sequence of the short type has homology with the N-terminal part of the other types. The short type and the N-terminal part of the other types of proteins in the natterin family can be assumed to consist of the lectin domain; however, the domain structure and function of the other parts cannot be ascertained yet. Nevertheless, some proteins in the natterin family—natterins from *T. nattereri* and Toxins I and II from *P. lineatus*—show toxicity, which implies that the C-terminal region of the proteins performs the function of a toxin. In line with this notion, it was reported that a part of the C-terminal region (residues 151–173) in a natterin-like protein from lamprey shares similarity with the 2-stranded sheet region of aerolysin^23^. This region in aerolysin is supposed to be important for membrane penetration and toxicity^24^. Furthermore, the C-terminal region (151–173) in the natterin-like protein has partial homology with the corresponding regions in natterins and Toxins I and II. Therefore, the same function can be hypothesized for the C-terminal region of the toxic proteins of the natterin family.

Recently, the structural analysis of the aerolysin-like protein Dln1 from zebrafish, which shares 24–29% sequence identity with the natterin proteins from *T. nattereri*, was reported^25^. Protein structure of Dln1 is composed of N-terminal region, assigned as “lectin module”, and C-terminal region, assigned as “aerolysin module”. The C-terminal regions of Dln1 and natterin proteins share homology between them, while the N-terminal regions do not (Supplementary Fig. S4). On the other hand, N-terminal region of the natterin proteins share the protein sequence of CGL1. Therefore, folding of CGL1 and the N-terminal region of proteins in the natterin family except Dln1 is still unknown.

Detailed amino acid sequence analysis showed that the sequence of CGL1 contains repeats divided between the first and second halves, with intra-sequence identity of 30% (Supplementary Fig. S5); each of the halves has 34–39% homology with the DM9 domain^26^ in the proteins from *Drosophila melanogaster*. The DM9 domain is found mainly in *Diptera* proteins, and sequence information on 170 proteins including the DM9 repeats has been deposited in the UniProt database^27^. Although the function of the DM9 domain in *Diptera* is not yet clear, the functional relation between CGL1 and the DM9 domain is intriguing.

### Expression of Recombinant CGL1

On the basis of the full-length sequence of CGL1 obtained from the database, we attempted gene cloning and expression of the recombinant CGL1 for further functional and structural analyses. The gene of CGL1 was cloned by PCR using primers designed from the gene and cDNA, prepared by reverse-transcription PCR using total mRNA isolated from the body of *C. gigas*. The sequence was confirmed to be identical to that in the database. The amplified DNA was ligated into the pET-3a vector at an appropriate site, and the resultant plasmid was introduced into *E. coli* BL21(DE3)pLysS cells. The transformed cells were cultivated, and expression of the recombinant CGL1 (wtCGL1) was induced with isopropyl-β-D-thiogalactopyranoside. After harvesting, disruption, and centrifugation of the recombinant *E. coli* cells, we performed purification of the recombinant CGL1 using the same approach as with native CGL1 (nCGL1) from *C. gigas*. Both types of purified CGL1s were used for further structural analysis as described below.

### Circular Dichroism (CD) Analysis of Native and Recombinant CGL1

CD spectra of nCGL1 and wtCGL1 in the presence and absence of mannose were acquired in the far UV region to examine the secondary structure and the effects of binding of mannose on the overall secondary structure. As shown in Supplementary Fig. S6, the shape of the spectra with the negative maximum at 216 nm indicates that CGL1 is mainly composed of β-sheets. The close similarity of CD spectra of CGL1s in the presence and absence of mannose implies that CGL1 binds mannose without a conformational change in the secondary structure.

### Crystallization of CGL1

To identify the structure of CGL1 as a novel lectin and the structural basis for its strict specificity for mannose, we carried out crystallization by the vapor diffusion method at 20 °C. As mentioned in *Methods*, 3 types of CGL1 solutions for crystallization were prepared: CGL1/FREE, purified from *C. gigas*, dialyzed against TBS, and concentrated to 5 mg/mL; CGL1/MAN, purified from *C. gigas*, undialyzed, and concentrated to 10 mg/mL; CGL1/MAN2, purified after heterogenous expression in *E. coli*, undialyzed, and concentrated to 10 mg/mL with 100 mM mannose. These 3 types of crystals were used for data collection. Data collection statistics are summarized in Table 2.

### Structure determination by S-SAD

After failing at initial screens to generate heavy atom derivatives by soaking heavy atoms into CGL1/MAN crystals, we tried to determine the CGL1 structure by using Sulfur Single-wavelength Anomalous Diffraction (S-SAD) experiments on the in-house Cu K-alpha source. The S-SAD method utilizes the slight anomalous dispersion effect of sulfur that often leads to difficulty in phase determination of a protein structure, even if the resolution of the X-ray diffraction is rather high (~1.5 Å). To overcome this difficulty, we enforced high-redundancy data collection, which includes 3,000 diffraction images covering a total oscillation angle of 1500 degrees from one protein crystal, to obtain statistically processed data with sufficient accuracy for S-SAD. In addition, after determination of the initial phase, Non Crystallographic Symmetry (NCS) averaging with four molecules of CGL1 in the asymmetric unit was performed using the program PHENIX^28^. Electron density maps after phase improvements, including the NCS averaging, yielded high enough quality for tracing all residues of CGL1. In this way, structure determination of CGL1 by the S-SAD method on the in-house Cu K-alpha source was achieved by means of the two additional methods, *i.e*., collection of diffraction data with high redundancy and NCS averaging for phase improvement.

### Overall Structure of CGL1

**Figure 5:**
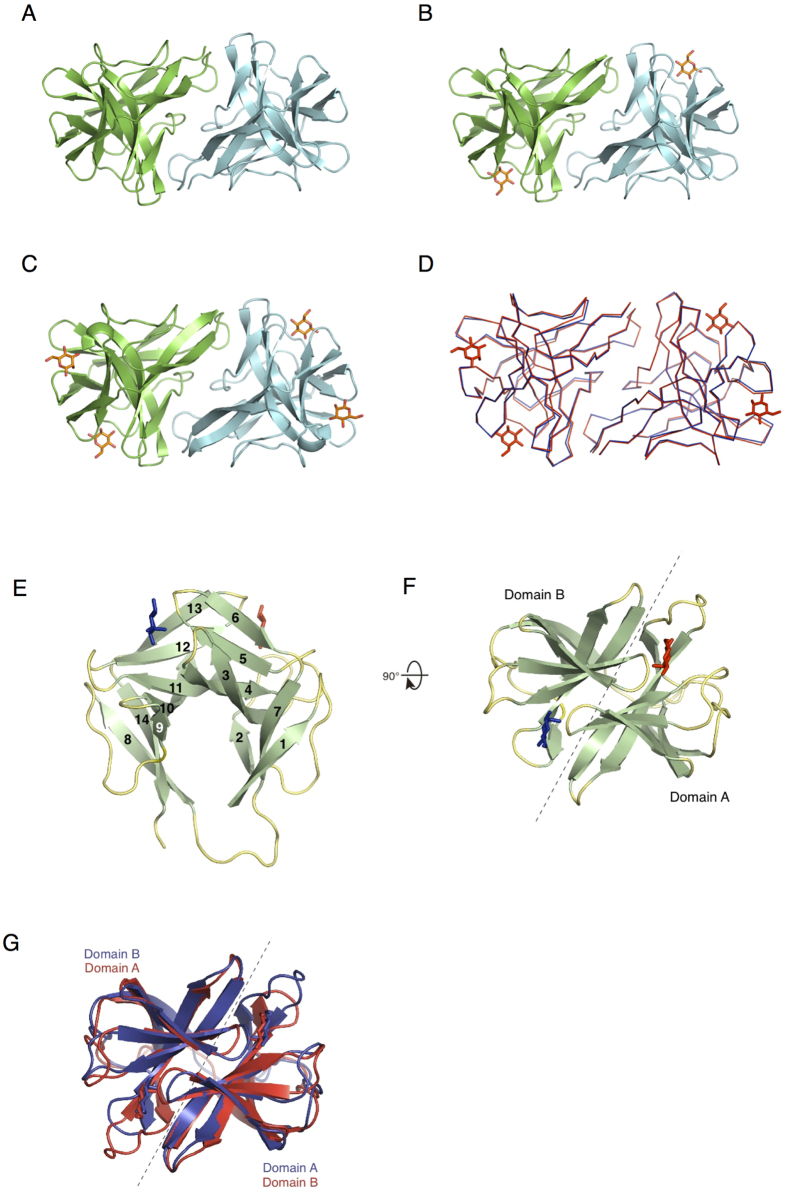
Crystal structure of CGL1. Crystal structures are shown in ribbon mode. (**A**) CGL1/FREE: CGL1 purified from *Crassostrea gigas*, dialyzed against TBS, and concentrated to 5 mg/mL; (**B**) CGL1/MAN: CGL1 purified from *C. gigas*, undialyzed, and concentrated to 10 mg/mL; and (**C**) CGL1/MAN2: CGL1 purified after heterogenous expression in *Escherichia coli*, undialyzed, and concentrated to 10 mg/mL with 100 mM mannose. Each protomer is shown in a different color. Mannose molecules that are bound to CGL1 are shown as orange stick figures. Each protomer of the dimer structure of CGL1/FREE and CGL1/MAN is related by 2-fold crystallographic symmetry. The dimer structure of MAN2 is an asymmetric unit. (**D**) Superposed structures of CGL1/FREE (blue) and CGL1/MAN2 (red). Side (**E**) and top (**F**) views of CGL1 domains are shown as a ribbon diagram. The numbering in panel *E* is the same as in Supplementary Fig. S8. Mannose molecules bound to sites A and B are shown as red and blue stick figures, respectively. (**G**) Superposed structures of domains A and B, which are related according to pseudo-2-fold symmetry. Each domain is colored differently.

Three types of crystal structures of CGL1 are shown in Fig. 5. Each type of structure contained a different number of mannose molecules per protomer (CGL1/FREE, 0; CGL1/MAN, 1; CGL1/MAN2, 2; Fig. 5A–C). We presumed that these differences were caused by the different mannose concentrations under the crystallization conditions (CGL1/FREE: dialyzed against TBS; CGL1/MAN: undialyzed during the purification process; and CGL1/MAN2: the buffer contained 100 mM mannose during crystallization) and different affinity of the 2 sites in CGL1 for mannose: high- and low-affinity sites A and B, respectively. A mannose bound to site A, the high-affinity site, in the CGL1/MAN structure likely came from the elution buffer, i.e., 100 mM mannose in TBS during affinity chromatography, and was retained during gel filtration and crystallization processes. Crystal structures of CGL1/FREE and CGL1/MAN include 4 CGL1 molecules in an asymmetric unit, in which intermolecular interactions among the CGL1 molecules are unequal and relatively weak (interaction surface areas are 28–284 Å^2^). On the other hand, intermolecular interactions with the next one related to the crystallographic 2-fold symmetry are equal and substantial (the interaction surface area is 1159 Å^2^, and it includes 15 hydrogen-bonding interactions). The strong intermolecular interactions were also confirmed in the CGL1/MAN2 structure’s asymmetric unit. Therefore, it is appears that the biological unit of CGL1 is a dimer similar to dimeric CGL1/MAN2 in the asymmetric unit (Fig. 5A–D). Computational analysis of the multimeric state in the PISA software^29^, Dynamic Light Scattering (DLS) and Small Angle X-ray Scattering (SAXS) analyses also showed an approximate dimeric structure in an aqueous solution (Supplementary Tables S2 and S3, Fig. S7). The intermolecular interactions in the dimeric structure consist of 23 residues per monomer, with hydrogen bonding and hydrophobic interactions (Supplementary Fig. S8). The amino acid residues involved in the dimerization are not strongly conserved in the natterin family.

The monomeric structure of CGL1 consists of 2 domains, A and B, including seven β-sheets per domain (Fig. 5E,F). Each of the domain structures is comparable and related to pseudo-2-fold symmetry (Fig. 5G, Supplementary Fig. S9). The structure of CGL1 is not recognizable in known lectin structures, suggesting that this is a novel lectin fold, which we named the “N (natterin)-type lectin fold.” The structure does not contain any inter- or intrachain disulfide bonds, whereas 4 mannose-binding sites of the CGL1 dimer are far away from one another; this feature is common in other lectin families. Because one of the likely functions of invertebrate lectins is to aggregate foreign microorganisms by binding to their surface carbohydrates, such arrangement of the binding sites may be advantageous.

During comparison of the structures of nCGL1 and wtCGL1, obvious difference was confirmed in an N-terminal residue, Ala2, which was not modified in the crystal structure of wtCGL1, but in nCGL1, it was modified with an acetyl group (Supplementary Fig. S10). The acetyl group showed intramolecular hydrophobic interactions with Tyr70, Pro71, Ala74, and Leu75 that are expected to stabilize these residues. The nCGL1 is more thermally stable than wtCGL1 expressed in *E. coli* (Supplementary Fig. S10). Therefore, it is likely that the N-terminal modification by the acetyl group stabilizes the whole nCGL1.

### Structure of the Mannose-Binding Site of CGL1

**Figure 6:**
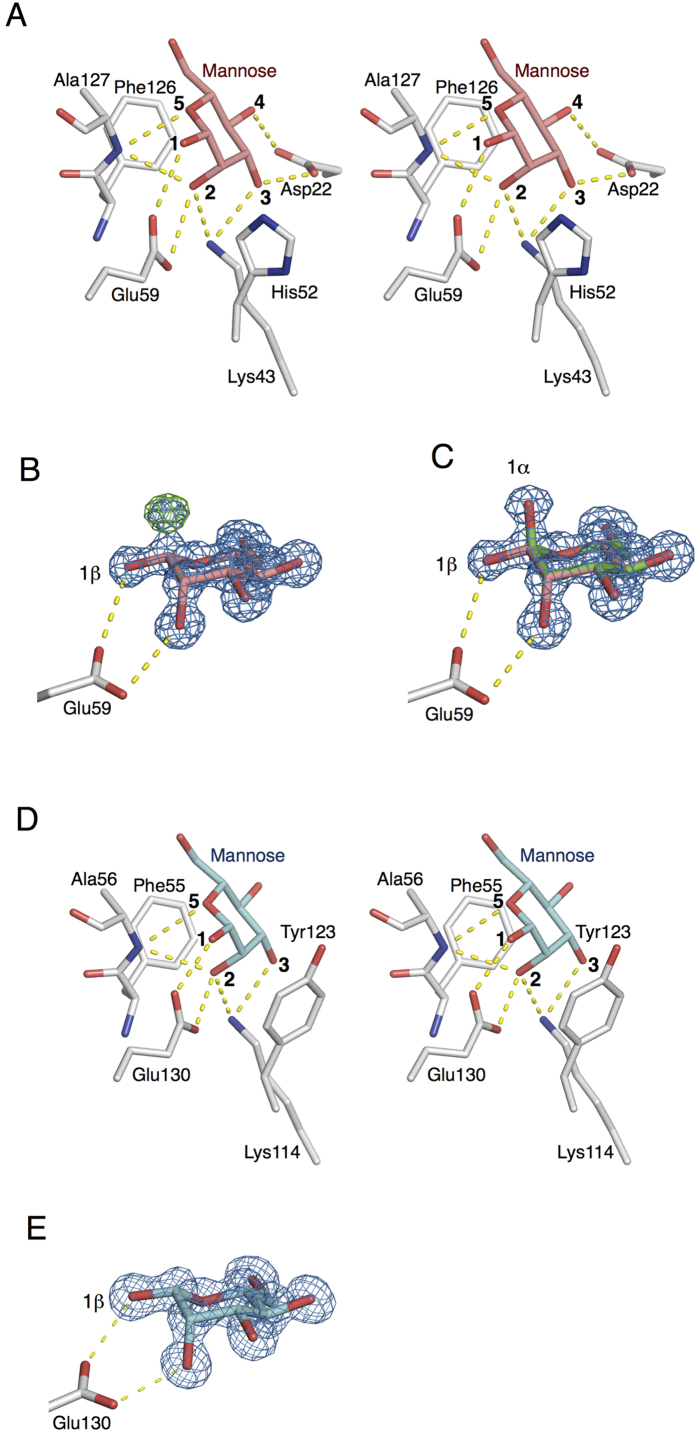
Illustration of the interactions between mannose and residues at sites A and B of CGL1. Mannose and residues at site A (panels A–C) and site B (panels D,E) are shown as stick figures. The dotted lines denote hydrogen bonds. *A*, a stereo view of mannose and the interacting residues at site A. *B*, β-mannose and the interacting Glu59 at site A with 2*F*_o_-*F*_c_ and *F*_o_-*F*_c_ electron density maps. *C*, superposition of anomeric mannoses and interacting Glu59 with the 2*F*_o_-*F*_c_ electron density map. *D*, a stereo view of mannose and the interacting residues at site B. *E*, mannoses and interacting Glu130 at site B with the 2*F*_o_-*F*_c_ electron density map. The 2*F*_o_-*F*_c_ and *F*_o_-*F*_c_ electron density maps are blue and green and are contoured at 1.5σ and 4σ, respectively.

Two mannose-binding sites, A and B, located on boundaries between the domains related to the pseudo-2-fold axis, are composed of residues from both domains (Figs 5E–G and 6). The mannose that is bound to site A is held by 8 hydrogen bonds with the side chain oxygens of Asp22 and Glu59, a nitrogen of Lys43, and the main-chain amide nitrogen of Ala127, where the hydroxyl and oxygen groups at positions 1, 2, 3, 4, and 5 of the β-mannose residues are bound on the other side. In addition, the binding of the mannose was further stabilized by stacking interactions between the hydrophobic parts of the mannose residue and side chains of His52 and Phe126 (Fig. 6A). These complicated interactions with a monosaccharide make it possible to achieve the specificity for mannose. During refinement of CGL1/MAN2 with β-mannose at site A, an obvious positive peak at a position corresponding to 1-OH group of α-mannose was confirmed on the *F*_o_-*F*_c_ map (Fig. 6B). After refinement of multiple structures with α- and β-anomeric mannose molecules, the positive peak on the *F*_o_-*F*_c_ map disappeared, and the proper shape (as multiple structures) appeared on the 2*F*_o_-*F*_c_ map, indicating that both anomeric mannoses are bound to site A (Fig. 6C). According to comparison of hydrogen-binding interactions of α- and β-mannoses within site A, α-mannose lacks the hydrogen bonding interaction between the 1-OH residue and Glu59 because of the steric arrangement.

The other mannose-binding site holds a mannose molecule via hydrogen bonds with the side chain nitrogen of Lys114, oxygen of Glu130, and a main-chain amide nitrogen of Ala56, in addition to hydrophobic interactions with Phe55 and Tyr123 (Fig. 6D). The binding mechanism for mannose at site B is mostly similar to that at site A except for Asp22, which has 2 hydrogen-binding interactions with the 3- and 4-OH groups of mannose at site A (does not correspond to any residues at site B). The lack of the 2 hydrogen-bonding interactions introduced by Asp22 is probably responsible for the weaker binding affinity for mannose at site B compared to site A. Aside from Asp22, the other differences, His52 of site A and Tyr123 of site B, are expected to influence the binding affinity, although the properties of these 2 residues are similar and involve mannose’s binding to a hydrophobic region. The number of bound mannose and αMeMan molecules per subunit of CGL1 was estimated to be 0.88 ± 0.35 (nCGL1) and 1.30 ± 0.53, respectively, by ITC analysis, whereas 2 mannose molecules were found to be bound to one subunit of CGL1 in the crystal structure of CGL1/MAN2. These results may be due to the higher concentration of mannose used for crystallization (100 mM), which enabled mannose to bind to site B which has relatively weak affinity for mannose. A subtle difference in the binding preference of sites A and B was also found in the crystal structure. While mixed electron densities of α- and β-mannose are seen in site A, only β-mannose is seen in site B in the CGL1/MAN2 structure (Fig. 6E), suggesting that site B preferably binds β-mannose. On the other hand, mannobiose (Manα1-2Man), a disaccharide unit at the terminal ends of HMTGs, was found to bind to the nCGL1 monomer with relatively high affinity and binding ratio (*K*_a_ = 6.00 ± 0.99 and *n* = 1.65 ± 0.09) (Table 1). This also indicates that site B has a lower affinity for mannose compared to site A, and can only be occupied by carbohydrates with higher affinity, such as mannobiose.

**Figure 7:**
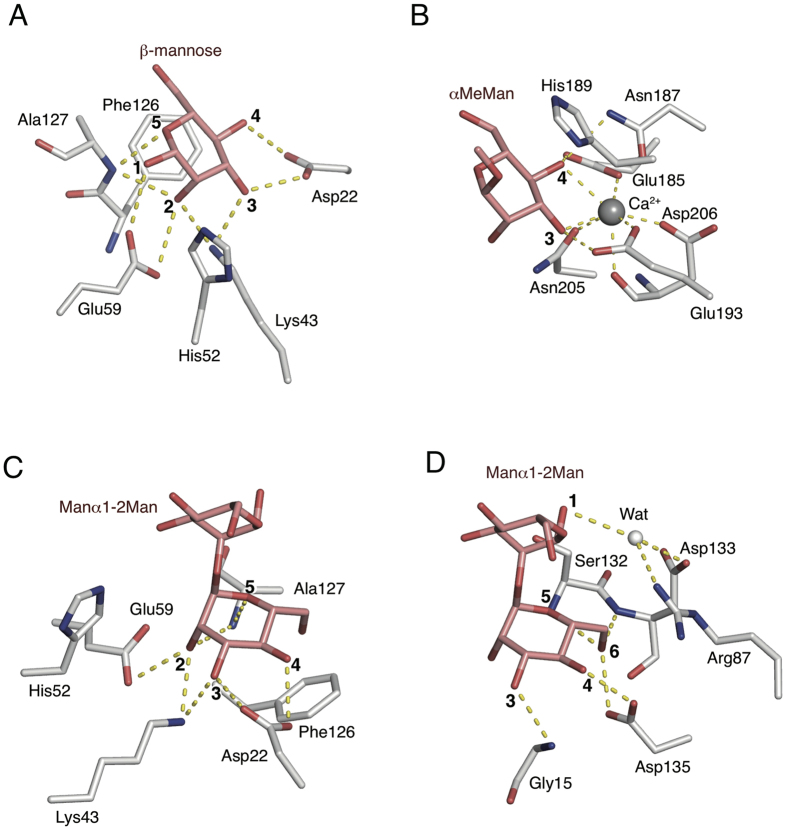
Structural comparison of residues for binding of mannose in CGL1 and other mannose-binding lectins. Stick models for the interactions of mannose with CGL1(**A**), α-Me-Mannoside with MBP-A from rat serum (**B**), binding model of CGL1 and mannobiose (**C**), and Dln1and mannobiose (**D**). Ca^2+^ ions are indicated in gray spheres. Mannose, the mannose-derivative, and the residues are shown as stick figures. The dotted lines denote hydrogen bonds. Residues without dotted lines have hydrophobic interactions with the sugars.

In comparison with the structures of CGL1-mannose (site A) and MBP-A-αMeMan complexes (PDB code; 1KWU [MBP-A from rat serum])^6^,^30^, it is revealed that a number of OH groups of mannose having hydrogen bonds with residues in CGL1 is significantly larger than those of αMeMan in MBP-A. Specifically, residues in CGL1 have hydrogen bonds to 1-, 2-, 3-, 4-, and 5-OH groups of mannose, while that of MBP-A have hydrogen and Ca^2+^ coordination bonds to 3- and 4-OH groups of αMeMan (Fig. 7). The MBPs, which have typical characteristics similar to that of the mannose-binding lectins, have a broad carbohydrate specificity, including their binding to mannose GlcNAc and fucose. These sugars have a common feature as vicinal equatorial hydroxyl groups in the stereochemistry, and are referred to herein as “Man-type”ligands. Residues and Ca^2+^ ions in MBPs-αMeMan complex structures has hydrogen and coordinate bonds only with the common feature of sugar, i.e. 3- and 4-OH groups of mannose derivatives, in the (Fig. 7B). Thus, the limited interaction in MBPs for sugars enables to show the broad carbohydrate specificity for the Man-type ligands. In contrast, orientation of the residues (which have complex hydrogen-bonding interactions with 1-, 2-, 3-, 4-, and 5-OH groups of mannose) at the CGL1 site A would enable the high specificity for binding to the mannose monomer and terminal mannoside residues in HMTGs.

We constructed a postulated binding model of mannobiose and CGL1 (Fig. 7C), in which the non-reducing mannoside residue of mannobiose was superimposed on α-mannose in CGL2/MAN2. This model shows that there is no steric crush between mannobiose and residues in CGL1. It is possible that some additional interactions between the reducing mannose residue and the binding site contribute to the higher affinity for this carbohydrate. A structural comparison with the lectin module of the Dln1-mannobiose complex^25^ is shown in Fig. 7D. Dln1 is one of the jacalin-related mannose-binding lectins^25^ with a β-prism fold, and specifically binds some HMTGs. In the structure of the Dln1-mannobiose complex, the residues in the binding site form hydrogen bonds with the 3-, 4-, 5-, and 6-OH groups of the non-reducing mannoside residue in mannobiose in addition to a water-mediated hydrogen bonding network with the reducing mannoside. Such interactions, including hydrogen bonds with more than one mannoside residue in the target oligosaccharides, would enable its specific binding to HMTGs. The structure of the N-terminal domain of natterin proteins is completely different from the lectin module in Dln1, while both domains might be involved in the pore-formation process through binding to the cell surface carbohydrate chains. Further investigation of the carbohydrate-recognition mechanism of CGL1 may provide useful information concerning the roles of these lectin domains in their pore-formation mechanism.

### Conclusion

In this study, we found that a novel lectin, which we named CGL1, purified from *C. gigas* has strict specificity for mannose monomer and HMTGs. A glycoconjugate microarray and FAC analysis demonstrated that CGL1 has binding specificity for mannose monomer, some of the HMTGs, while some unknown oligosaccharides without mannoside residues could also be recognized by CGL1 as other potential targets. We analyzed the primary structure of the CGL1, and it does share homology with the natterin family and DM9 domain, whose function is not known yet. Crystal structure of CGL1 shows its unique folding including 2 domains. Complex structures of CGL1 with mannoses show precise and unique interactions with a bound mannose, thereby explaining the strict specificity. The strict specificity of CGL1 may also hold great promise as a research tool and for clinical applications^31^, for example, a protein tag expressed as a CGL1-fusion protein that can be expressed in *E. coli* and purified based on the affinity for mannose-conjugated resin, for the detection of defects in glycosylation that appear in abnormal mammalian cells, and as a drug against viruses and bacterial pathogens.

## Experimental Procedures

### Animals

Oysters (*Crassostrea gigas*) that were cultured in the Seto Inland Sea were purchased from a local dealer. The shells were removed, and the bodies were stored at −20 °C.

### Purification of CGL1 from C. gigas

CGL1 was purified from *C. gigas* by affinity chromatography using a column with resin-immobilized mannose, which was prepared by conjugating mannose with a Cellulofine gel (Seikagaku Kogyo) using the cross-linking reagent divinyl sulfone^32^. Shelled and frozen bodies of *C. gigas* (500 g) were suspended in 600 mL of TBS (Tris-buffered saline; 10 mM Tris-HCl pH 7.6, 150 mM NaCl) and disrupted in a blender, followed by centrifugation at 9,500 × *g* for 30 min. The supernatant was applied to the mannose-Cellulofine column (5 mL) pre-equilibrated with TBS. After washing with this buffer, we eluted CGL1 with 100 mM mannose in TBS. The protein was further purified by gel filtration on a HiLoad 26/60 Superdex 200 prep grade column (GE Healthcare) equilibrated with TBS and was then eluted at the flow rate of 2.5 mL/min using an ÄKTAprime plus apparatus (GE Healthcare). The purified protein solution was concentrated to 3 mg/mL and dialyzed at 4 °C against TBS for removal of mannose. For crystallization of the mannose complexes with CGL1, the purified protein solution was concentrated to 10 mg/mL, without subsequent dialysis. Approximately 10 mg of CGL1 was obtained from 500 g of the shelled bodies.

### Titration Microcalorimetry

Sugar specificity and thermodynamic parameters were measured by isothermal titration calorimetry (ITC) using a MicroCal iTC_200_ microcalorimeter (GE Healthcare). The titration was carried out at 25 °C. The ligands (mannose, methyl-α-mannoside (αMeMan), galactose, glucose, rhamnose, N-acetylglucosamine (GlcNAc), N-acetylgalactosamine (GalNAc), galactosamine, N-acetylneuraminic acid (NeuNAc), fucose, methyl-α-galactoside (αMeGal), methyl-β-galactoside (βMeGal), and mannobiose (Manα1-2Man)) and CGL1 were dissolved in TBS. Titration of CGL1 binding was performed in a cell in a volume of 200 μL by means of 20 injections of 2 μL ligand at 2-min intervals, with simultaneous stirring at 1,000 rpm. CGL1 and ligand solutions were used at concentrations of 0.30 mM (4.6 mg/ml) and 8.9 mM. A control experiment was carried out to measure the ligand dilution-related heat, which was subtracted from the ligand binding thermogram before data analysis.

### Glycoconjugate microarray

The sugar-binding specificity of CGL1 toward complex oligosaccharides was analyzed using a glycoconjugate microarray in accordance with a published method^33^. Briefly, glycoproteins and glycoside-polyacrylamide conjugates were dissolved in a spotting solution (Matsunami Glass) and spotted on a microarray-grade epoxy-coated glass slide (Schott AG) using a non-contact microarray-printing robot (MicroSys 4000; Genomic solutions). CGL1 was labeled with Cy3-NHS ester (GE healthcare) and incubated with the glycoconjugate microarray at 20 °C overnight. After washing, images were immediately acquired using a Bio-REX Scan 200 evanescent field-activated fluorescence scanner (Rexxam).

### Frontal affinity chromatogramphy (FAC)

FAC analysis was performed as described previously^34^. Briefly, CGL1 was immobilized on NHS-activated Sepharose 4 Fast Flow (GE Healthcare) at a concentration of 0.2 mg/mL and packed into a miniature column (inner diameter, 2 mm; length, 10 mm; bed volume, 31.4 μL; Shimadzu) and connected to an automated FAC system (FAC-2). A panel of pyridylaminated (PA) and p-nitrophenol (pNP) glycans were successively injected into the columns by the auto-sampling system, and elution was detected by fluorescence (excitation, 310 nm; emission, 380 nm) or absorbance at 280 nm. The elution front of each glycan relative to that of an appropriate control, referred to as V-V_0_, was then determined.

### Chemical Cleavage, Separation, and Sequencing of Peptides

The purified CGL1 was reduced with tri-*n*-butylphosphine in 7M guanidine-HCl, 10 mM EDTA, 0.5 M Tris-HCl pH 8.5. The reduced CGL1 was pyridylethylated with 4-vinyl pyridine at 25 °C for 4 h in the dark. The reduced and pyridylethylated CGL1 was chemically cleaved at the methionyl bonds with 1% CNBr in 70% (v/v) formic acid at 25 °C for 24 h by the method of Gross^28^,^35^,^36^. The peptides generated by the CNBr cleavage were separated by reverse-phase high-performance liquid chromatography (RP-HPLC) using HITACHI model L-6200 and L-4200 liquid chromatographs on a Wakosil-II 5C18 AR column (4.6 × 100 mm, Wako Pure Chemical Industries Ltd., Osaka, Japan). The column was equilibrated with solvent A (0.1% trifluoroacetic acid; TFA), and the peptides were eluted at the flow rate of 1 mL/min using a linear gradient of 0–100% solvent B (acetonitrile/water/TFA, 80:20:0.1 [v/v/v]) at room temperature. The separated fractions were collected for sequencing. Automated Edman degradation^37^,^38^,^39^,^40^ was performed using a gas phase protein sequencer (Shimadzu model PPSQ-21).

### Bioinformatics Analysis

Online sequence homology searches (www.ncbi.nlm.nih.gov) were performed using the blastp algorithm in nonredundant databases of the National Center for Biotechnology Information (NCBI)^28^,^29^,^41^,^42^. Sequence alignment was performed in the Clustal Omega software^43^,^44^.

### cDNA Cloning and Heterologous Expression of CGL1

Oligonucleotide primers were designed based on the DNA sequence of CGL1 that we determined (see *Results and Discussion*): CGL1-F1 5′-CATATGGCAGAGTGGGTATCAACGAC-3′ and CGL1-R1 5′-GGATCCCTAAATGACTTTATACAGAGCTT-3′. Total RNA was isolated from shelled *C. gigas* by using the NucleoSpin RNA II kit (MACHEREY-NAGEL GmbH & Co. KG, Germany). Reverse-transcription PCR was performed using the Prime script II First-Strand cDNA Synthesis Kit (Takara Bio Inc., Japan) with an oligo(dT) primer. Thermal cycling conditions were as follows. The reverse-transcription PCR mixture was incubated at 42 °C for 45 min for the reverse transcriptase reaction, followed by 30 cycles of PCR. The amplified fragment was cloned into the pTAC-2 vector using a kit (DynaExpress TA PCR Cloning Kit [pTAC-2], BioDynamics Laboratory Inc.) and sequenced to confirm the absence of PCR-related errors. The plasmid was digested with *Nde*I and *Bam*HI, and the resulting DNA fragment was ligated with the pET-3a vector (Novagen) that had been digested with *Nde*I and *Bam*HI to obtain the plasmid pET-3a-CGL1. The resultant plasmid was introduced into *Escherichia coli* BL21(DE3)pLysS cells, and the protein was induced with 0.4 mM isopropyl β-d-thiogalactopyranoside. After harvesting the cells, they were disrupted at 4 °C using a High-Intensity Ultrasonic Liquid Processor (Sonic & Materials, Inc.). The cell debris were removed by centrifugation at 10,000 × *g* for 15 min. The supernatant was applied to the mannose-Cellulofine column (5 mL) equilibrated with TBS. The column was washed with TBS, and the recombinant CGL1 was eluted with TBS containing 0.1 M mannose. The eluate was collected, concentrated, and dialyzed against TBS to remove the mannose bound to CGL1. The recombinant CGL1 was used for characterization along with the native CGL1 purified from *C. gigas*.

### Crystallization

Crystallization of free CGL1 and its mannose complexes was carried out by the vapor diffusion method. Two to 4 μL of the protein solution (CGL1/MAN (S-SAD), CGL1/MAN, and CGL1/MAN2: 10 mg/mL; CGL1/FREE: 5 mg/mL) in TBS (CGL1/MAN2: containing 100 mM mannose) were mixed with the same volume of a reservoir solution (CGL1/MAN(S-SAD) and CGL1/MAN: 0.2 M magnesium acetate, 0.1 M sodium cacodylate [pH 6.5], 20% [w/v] polyethylene glycol 8000; CGL1/FREE and CGL1/MAN2: 30% (w/v) polyethylene glycol 3000, 100mM CHES [pH 9.5]) and were subjected to vapor diffusion at 20 °C.

### Data Collection, Structure Solution, and Refinement

The data sets from CGL1/MAN(S-SAD) were collected in-house (MicroMax007 & R-AXIS IV_++_ [RIGAKU]) whereas those from the other crystals were collected using beamline BL-17A at the Photon Factory (KEK, Tsukuba, Japan). All crystals were frozen at 95K before data collection. An external solution of crystals CGL1/MAN(S-SAD) and CGL1/MAN was processed with Paratone-N (Hampton Research) before freezing. The data set from CGL1/MAN(S-SAD) was processed and scaled in the MOSFLM^35^,^45^ and SCALA software^37^,^46^, respectively, and the other data sets were processed and scaled in HKL2000^29^,^38^. The data sets of CGL1/MAN2 and the other crystals belonged to space groups *P*2_1_2_1_2_1_ and *P*2_1_, with 2 and 4 molecules per asymmetric unit, respectively. The CGL1/MAN(S-SAD) data set was used for phase calculation by the sulfur single-wavelength anomalous diffraction method (S-SAD) in the PHENIX software^28^,^43^. Phase improvement by density modification, including noncrystallography symmetry (NCS) averaging, was also performed in PHENIX^28^,^35^. The structure was built using the COOT software^37^,^39^ and refined in Refmac^29^,^41^, with 5% of the data set aside as a free set. During subsequent refinement, the CGL1/MAN(S-SAD) data set was replaced by the CGL1/MAN data set. The mannose models were fitted into the carbohydrate-binding sites according to the difference electron density map. The structures of CGL1/FREE and CGL1/MAN2 were solved by the direct Fourier synthesis with the model of CGL1/MAN and the molecular replacement method using CGL1/MAN, respectively. The molecular replacement was performed using the Molrep CCP4 suite^43^,^44^. Anisotropy refinements of the structures were performed at the final stage of the refinement. The refinement statistics are listed in Table 1. Superposition of CGL1/FREE and CGL1/MAN was performed in the CCP4 software SUPERPOSE^35^. All figures were produced using the PyMOL software (http://www.pymol.org). Secondary structure assignment was calculated in DSSP^37^. The interface surface area and assemblies of CGL1 were calculated using PISA^29^. Sequence alignment was performed in the Clustal Omega software^43^.

## Additional Information

**Accession codes:** The atomic coordinates and structural parameters of CGL1 were deposited in the Protein Data Bank, www.pdb.org (PDB codes: CGL1/FREE, 5ID8; CGL1/MAN, 5IDA; CGL1/MAN2, 5IDB). This work has been performed under the approval of the Photon Factory Program Advisory Committee (Proposal No. 2013G027).

**How to cite this article**: Unno, H. *et al*. Identification, Characterization, and X-ray Crystallographic Analysis of a Novel Type of Mannose-Specific Lectin CGL1 from the Pacific Oyster *Crassostrea gigas. Sci. Rep.* **6**, 29135; doi: 10.1038/srep29135 (2016).

## Supplementary Material

Supplementary Information

## Figures and Tables

**Table 1:** Thermodynamic characteristics of CGL1’s binding to carbohydrates.

CGL1	Sugar	*n*	*K*
			× 10^3^ M^−1^
nCGL1	Mannose	0.88 ± 0.35	2.00 ± 0.46
	α-MeMan	1.30 ± 0.53	1.49 ± 0.50
	Manα1-2Man	1.65 ± 0.09	6.00 ± 0.99

**Table 2:** Data collection and refinement statistics.

Crystal type	CGL1/MAN(S-SAD)	CGL1/FREE	CGL1/MAN	CGL1/MAN2
Data collection and processing statistics				
X-ray source	Rigaku	Photon factory	Photon factory	Photon factory
	Micromax007	BL-17A	BL-17A	BL-17A
Wavelength (Å)	1.5418	0.9800	0.9800	0.9800
Space group	*P*2_1_	*P*2_1_	*P*2_1_	*P*2_1_2_1_2_1_
Unit cell dimension (Å)				
*a* (Å)	39.5	39.6	39.7	57.3
*b* (Å)	58.7	58.7	58.8	57.9
*c* (Å)	108.3	107.9	108.5	80.4
β (°)	93.7	93.8	93.7	90.0
Resolution (Å)	30.85–1.50	50.0−1.10	50.0−1.1	23.5–1.00
	(1.58–1.50)^a^	(1.12−1.10)	(1.12−1.10)	(10.5–1.00)
Total reflections	1,238,131	709,916	624,618	1,001,962
Unique reflections	71,137	173,353	160,510	131,088
*I*/σ*I*	46.7 (12.1)	14.9 (2.8)	17.0 (7.5)	16.4 (3.2)
Redundancy	17.4 (11.0)	4.1 (4.7)	3.9 (4.2)	7.6 (4.6)
Completeness (%)	89.7 (50.8)	86.6 (99.0)	79.6 (99.9)	89.0 (53.0)
*R*_merge_^b^ (%)	3.9 (13.4)	10.9 (50.3)	9.6 (26.7)	7.3 (30.3)
Refinement statistics				
Resolution		30.9−1.10	17.87–1.10	22.4–1.00
Protein molecules per asymmetric unit		4	4	2
Mannose molecules per asymmetric unit		0	3	4
Protein atoms		4360	4360	4328
Ligand atoms		0	36	174
Water molecule		698	711	486
B-factors				
Protein		21.6	24.3	9.0
Ligand atoms		–	30.2	9.8
*R*_work_/*R*_free_(%)		16.7/22.0	16.4/22.2	12.3/14.5
Root mean square deviations				
Bond lengths (Å)		0.026	0.028	0.023
Bond angles (°)		2.289	2.447	2.053

^a^The values in parentheses correspond to the highest-resolution shell.

^b^*R*_merge_ = 100Σ|*I* − <*I*>|/Σ *I*, where *I* is the observed intensity and <*I*> is the average intensity of multiple observations of symmetry-related reflections.
