# Supplementary material for: Identification, Characterization, and X-ray Crystallographic Analysis of a Novel Type of Mannose-Specific Lectin CGL1 from the Pacific Oyster Crassostrea gigas
Source: Sci Rep. 2016 Jul 5;6:29135. doi: 10.1038/srep29135 (PMC4932603; doi:10.1038/srep29135)
Supplement: Supplementary Information [file srep29135-s1.pdf]

Supplemental Information

Identification, Characterization, and X-ray Crystallographic Analysis of a Novel Type of Mannose-Specific Lectin CGL1 from the Pacific Oyster *Crassostrea gigas*

Hideaki Unno, Kazuki Matsuyama, Yoshiteru Tsuji, Shuichiro Goda, Keiko Hiemori, Hiroaki Tateno, Jun Hirabayashi, and Tomomitsu Hatakeyama

SUPPLEMENTAL RESULTS

Estimation of Assembly by Measurements of SAXS and DLS. To confirm our estimate of the dimer as a biological unit by means of crystallographic analysis, we performed measurements of SAXS and DLS of CGL1s in a solution. Average diameters that were calculated from SAXS and DLS measurements (Supplementary Tables S2 and S3, respectively) are approximately equal to those of the dimeric form according to the crystallographic analysis (30 × 40 × 60 Å). In addition, the experimental curve of CGL1s in a Kratky plot matched well the theoretical curve calculated from the dimer structure but was obviously different from the theoretical curves calculated from monomer and tetramer models of CGL1 (Supplementary Fig. S7 online). These results on SAXS and DLS measurements (indicating that the assembly of CGL1 in a solution is the dimer) are in agreement with the picture obtained from the crystallographic analysis. As for the influence of mannose binding, there was no significant difference in the average diameter calculated from the SAXS and DLS data (Supplementary Tables S2 and S3) and from Guinier and Kratky plots between CGL1 in a solution with and without mannose. This similarity means that the binding of mannose to CGL1 does not cause any change in the multimeric state of CGL1 in a solution.

SUPPLEMENTAL EXPERIMENTAL PROCEDURES

Circular Dichroism (CD) Measurements. Far-UV CD measurements of the proteins were performed in the range of 200–250 nm on a J-720 spectropolarimeter (JASCO). The spectra were acquired using a quartz cell of 1-mm path length at 20 °C in TBS in the presence or absence of 10 mM mannose or 10 mg/mL mannan. Protein concentrations in the measurements of native (nCGL1) and wild type (wtCGL1) CGL1s were 0.54 mg/ml and 0.28 mg/ml, respectively.

Dynamic Light Scattering (DLS). Solutions of native and recombinant CGL1 in the presence and absence of 100 mM mannose were made: 1 mg/mL (native) and 6 mg/mL (recombinant) in TBS. The samples were analyzed in small-volume plastic cuvettes using a Zetasizer Nano ZS instrument (Malvern). Particle diameters were recorded as a frequency distribution curve, and the average diameter was calculated.

Small-Angle X-ray Scattering (SAXS). The SAXS measurements were taken on the beamline BL-10C at the Photon Factory (Tsukuba, Japan). The wave length of 1.488 Å and the specimen-to-detector distance of 958.8 mm were used, and all measurements were carried out at 25 °C in a temperature-controlled cell holder. The duration of the SAXS analysis of native and recombinant (wt) CGL1 (concentration ~3 mg/mL) was 300 and 60 s, respectively. The radius of gyration (R_g), forward scattering intensity (normalized to the protein concentration, $J(0)/C$), and maximum particle dimension (D_{max}) were then calculated. The calculation of the SAXS profile of the X-ray structure of CGL-1 (PDB code 4YJ8) was carried out with the program CRYSOL [1].

Measurement of Thermal Stability of the CGL1 using Sugar-Polyamidoamine Dendrimer Conjugates (Sugar-PDs). For preparation of PD conjugated with carbohydrates, we used a method described previously [2]. For measurements of the carbohydrate-binding activity of the CGL1, mannotriose-PD was prepared in the same way. The carbohydrate-binding activity was evaluated according to the increase in Rayleigh scattering of the CGL1 solution after addition of various sugar-PD solutions as a function of the complex formation. After recording the initial scattering intensity at 420 nm with the lectin solution (nCGL1, 30 μg/mL, 1 mL; wtCGL1, 6.2 μg/mL, 1 mL) in TBS using a Model F-3010 Fluorescence Spectrophotometer (Hitachi) at 20 °C, we added small volumes of a sugar-PD solution to the same buffer and recorded the changes in the scattering intensity. For measurement of thermal stability, CGL1 was preincubated at a series of temperatures for 15 min. After mixing the CGL1s and mannotriose-PD at 20 °C in TBS, we recorded the scattering intensity at the excitation and emission wavelength, 420 nm. For analysis of the effects of heterogenous expression in *E. coli*, we performed the measurements with both types of CGL1s, native CGL1 purified from *C. gigas* and the wild-type (recombinant) protein expressed in *E. coli*.

SDS-PAGE. This procedure was carried out according to the method of Laemmli [3]. The samples were heated in the presence or absence of β-mercaptoethanol for 4 min at 95 °C before loading onto the gel. The proteins on the gels were visualized with Coomassie Brilliant Blue R-250.

Hemagglutination Assays. These assays were performed in 96-well U-bottom plates. Fifty microliters of a 2-fold dilution of purified CGL1 (2.0 mg/mL) in TBS was mixed with 50 μL of a 5% suspension (in TBS; v/v) of rabbit, horse, sheep, bovine, or chicken erythrocytes. The plate was incubated at room temperature for 1 h, and the formation of a sheet (agglutination-positive result) or a dot (agglutination-negative result) was monitored.

SUPPLEMENTAL TABLES AND FIGURES

Supplementary Table S1. List of oligosaccharides for glycoconjugate microarray analysis.

Number	Glycans
1	Fuca1-PAA
**2**	Fuca1-2Galb1-PAA
3	Fuca1-3GlcNAcb1-PAA
**4**	Fuca1-4GlcNAcb1-PAA
5	Fuca1-2Galb1-3GlcNAcb1-PAA
**6**	Fuca1-2Galb1-4GlcNAcb1-PAA
7	Fuca1-2Galb1-3GalNAca1-PAA
**8**	GalNAca1-3(Fuca1-2)Galb1-4GlcNAcb1-PAA
9	Gala1-3(Fuca1-2)Galb1-4GlcNAcb1-PAA
**10**	Galb1-3(Fuca1-4)GlcNAcb1-PAA
11	(3OSO$_3$)Galb1-3(Fuca1-4)GlcNAcb1-PAA
**12**	Fuca1-2Galb1-3(Fuca1-4)GlcNAcb1-PAA
13	Galb1-4(Fuca1-3)GlcNAcb1-PAA
**14**	Fuca1-2Galb1-4(Fuca1-3)GlcNAcb1-PAA
15	Neu5Aca2-PAA
**16**	Neu5Gca2-PAA
17	Neu5Aca2-8Neu5Aca2-PAA
**18**	Neu5Aca2-8Neu5Aca2-8Neu5Aca2-PAA
19	Neu5Aca2-3Galb1-3GlcNAcb1-PAA
**20**	Neu5Aca2-3Galb1-4Glcb1-PAA
21	Neu5Aca2-3Galb1-4GlcNAcb1-PAA
**22**	Neu5Aca2-3Galb1-3(Fuca1-4)GlcNAcb1-PAA
23	Neu5Aca2-3Galb1-4(Fuca1-3)GlcNAcb1-PAA
**24**	Neu5Aca2-6Galb1-4Glcb1-PAA
25	Fetuin (Complex-type N-glycans and O-glycans)
**26**	a1-acid glycoprotein (Complex-type N-glycans-)
27	Transferrin (Complex-type N-glycans)
**28**	Porcine thyroglobulin (Complex and high-mannnose-type N-glycans)
29	Galb1-PAA
**30**	(3OSO$_3$)Galb1-PAA
31	GalNAca1-3Galb1-PAA
**32**	Galβ1-4Glcb1-PAA
33	Galb1-3GlcNAcb1-PAA
**34**	(3OSO$_3$)Galb1-3GlcNAcb1-PAA
35	Galb1-4GlcNAcb1-PAA
**36**	(3OSO$_3$)Galb1-4GlcNAcb1-PAA
37	Galb1-4(6OSO$_3$)GlcNAcb1-PAA
**38**	(6OSO$_3$)Galb1-4GlcNAcb1-PAA
39	GalNAcb1-PAA
**40**	GalNAcb1-3GalNAcb1-PAA
41	GalNAcb1-4GlcNAcb1-PAA
**42**	GalNAcb1-4Galb1-4Glcb1-PAA
43	Asialo fetuin (Desialylated complex-type N- and O-glycans)
**44**	Asialo a1-acid glycoprotein (Desialylated complex-type N-glycans)
45	Asialo transferrin (Desialylated complex-type N-glycans)
**46**	Asialo porcine thyroglobulin (Desialylated complex-type N-glycans, high-mannose-type N-glycans)
47	GlcNAcb1-PAA
**48**	(6OSO$_3$)GlcNAcb1-PAA

Number	Glycans
51	Agalacto transferrin (Agalactosylated complex-type N-glycans, high-mannose-type N-glycans)
**52**	Ovomucoid (Complex-type N-glycans)
53	Ovoalbumin (Hybrid-type N-glycans)
**54**	Mana1-PAA
55	Manb1-PAA
**56**	(6OPO$_4$)Mana1-PAA
57	Yeast invertase (High mannose-type N-glycans)
**58**	GalNAca1-PAA
59	Galb1-3GalNAca1-PAA
**60**	Galb1-3(GlcNAcb1-6)GalNAca1-PAA
61	GlcNAcb1-3GalNAca1-PAA
**62**	GlcNAcb1-3(GlcNAcb1-6)GalNAca1-PAA
63	GalNAca1-3GalNAcb1-PAA
**64**	GlcNAcb1-6GalNAca1-PAA
65	Gala1-3GalNAca1-PAA
**66**	(3OSO$_3$)Galb1-3GalNAca1-PAA
67	Galb1-4GlcNAcb1-3GalNAca1-PAA
**68**	Asialo bovine submaxillary mucin (Tn)
69	Asialo human glycophorin MN (T)
**70**	Neu5Aca2-6GalNAca1-PAA
71	Neu5Gca2-6GalNAca1-PAA
**72**	Neu5Aca2-3Galb1-3GalNAca1-PAA
73	Galb1-3(Neu5Aca2-6)GalNAca1-PAA
**74**	Bovine submaxillary mucin (Sialyl Tn)
75	Human glycophorin (Disialyl T and sialyl Tn)
**76**	Gala1-PAA
77	Gala1-2Galb1-PAA
**78**	Gala1-3Galb1-PAA
[illegible]	Gala1-3Galb1-4Glcb1-PAA
**80**	Gala1-3Galb1-4GlcNAcb1-PAA
81	Gala1-4Galb1-4GlcNAcb1-PAA
**82**	Gala1-6Glcb1-PAA
83	Glca1-PAA
**84**	Glcb1-PAA
85	Glca1-4Glcb1-PAA
**86**	Hyaluronic acid-BSA
87	Chondoroitin Sulfate A-BSA
**88**	Chondoroitin Sulfate B-BSA
89	Heparan Sulfate-BSA
**90**	Heparin-BSA
91	Keratan Sulfate-BSA
**92**	Rhamnosea1-PAA
93	*S. cerevisiae* mannan
**94**	*C.albicans* mannan
95	Zymosan
**96**	GlcNAcb1-4GlcNAcb1-PAA
97	-
**98**	-

Supplementary Table S2. Structural parameters of CGL1 according to small-angle X-ray scattering.

	R_g (Å)	D_{max} (Å)
nCGL1	21.6	73.9
nCGL1+ mannose	20.7	72.3
wtCGL1	21.6	69.0
wtCGL1 + mannose	20.3	64.8

nCGL1: native CGL1, wtCGL1: wild-type recombinant CGL1 expressed in *Escherichia coli*.

Supplementary Table S3. Dynamic light scattering estimates for the diameters of CGL1.

	Average diameter (nm)
nCGL1	5.3
nCGL1 + mannose	5.3
wtCGL1	5.5
wtCGL1 + mannose	6.0

nCGL1: native CGL1, wtCGL1: wild-type recombinant CGL1 expressed in *Escherichia coli*.

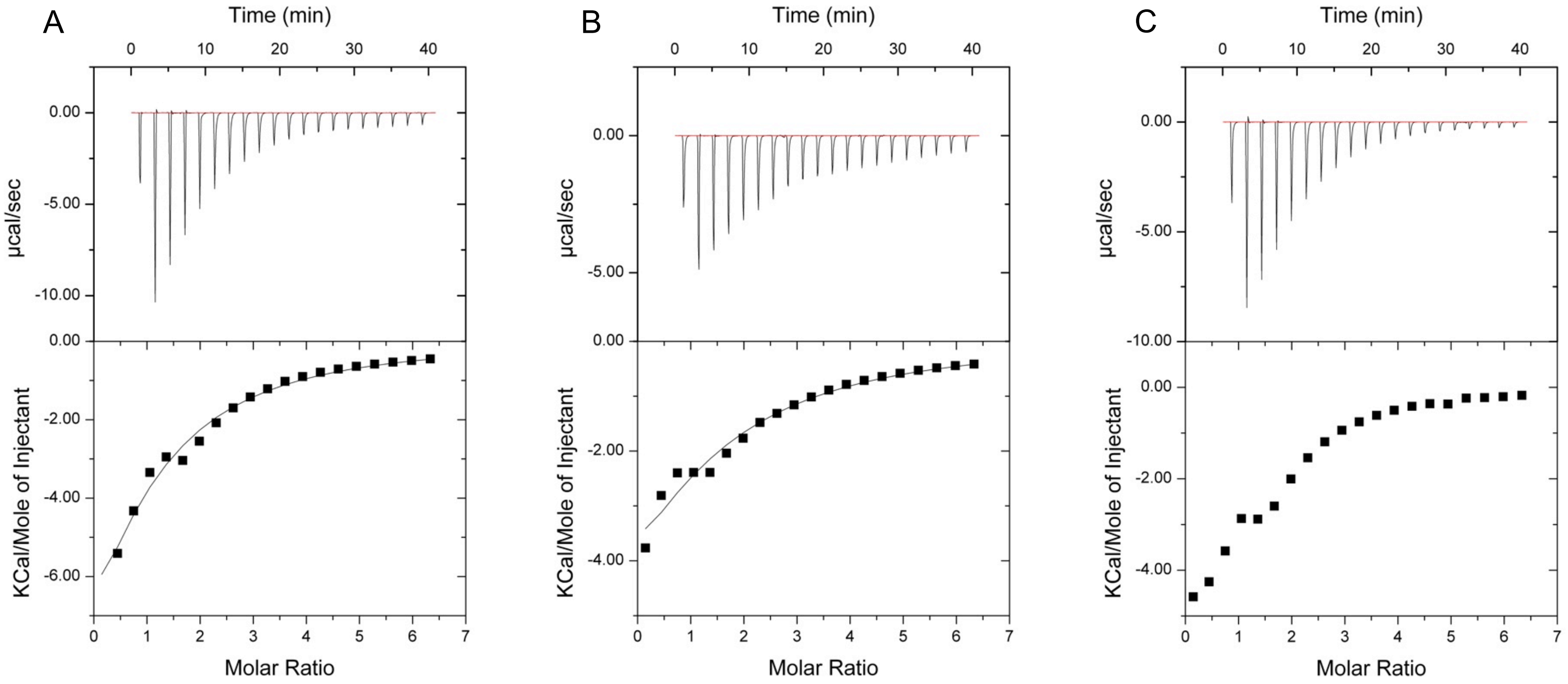

Supplementary Figure S1.

Supplementary Figure S1. Titration calorimetry results on CGL1 with mannose and methyl-α-mannoside. *A*, mannose (8.9 mM) in CGL1 (0.30 mM). *B*, methyl-α-mannoside (8.9 mM) in CGL1 (0.30 mM). *C*, mannobiose (Manα1-2Man) (8.9 mM) in CGL1 (0.30 mM). Top: the data from 20 automatic injections of 2 μL of mannose or methyl-α-mannoside or mannobiose into a CGL1-containing cell. Bottom: a plot of the total heat released as a function of ligand concentration for the titration shown above (squares). The solid line represents the best least square fit for the resulting data.

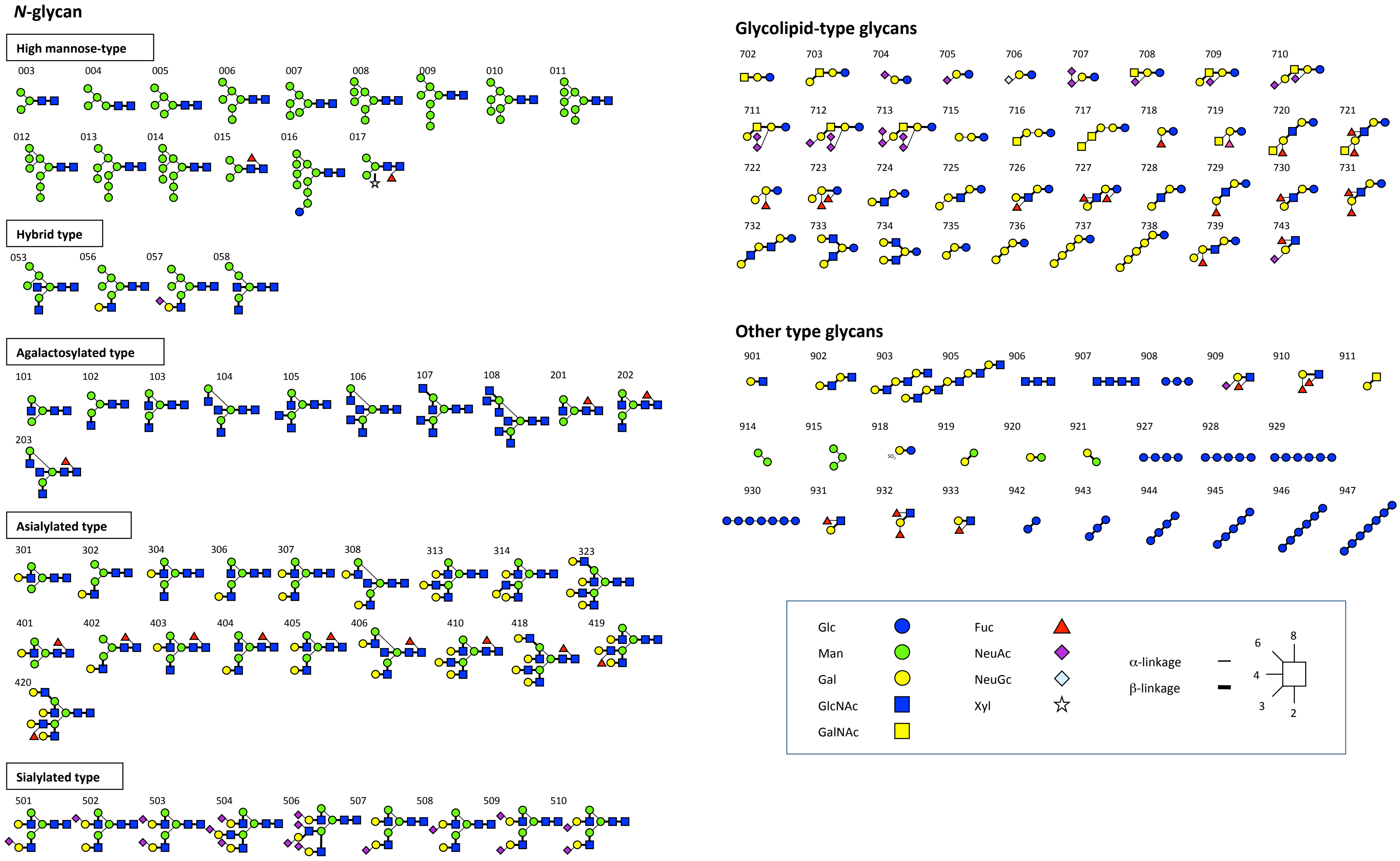

Supplementary Figure S2.

Supplementary Figure S2. Structures of PA and pNP glycans used in the frontal affinity chromatography.

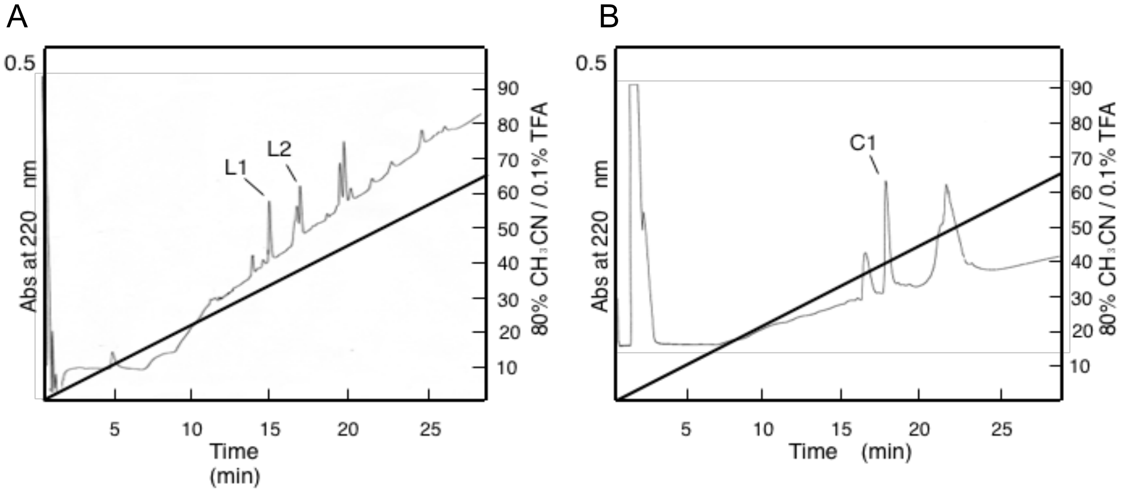

Supplementary Figure S3.

Supplementary Figure S3. Elution profiles from high-performance liquid chromatography (HPLC) of the peptides generated by enzymatic digestion and chemical cleavage of CGL1. The lysyl endopeptidase digest (*A*) and CNBr cleavage (*B*) on a Wakosil-II 5C18 AR column. The peptides were eluted with a gradient of acetonitrile into dilute aqueous trifluoroacetic acid (TFA).

```
CGL1          ------------------------------------------------------------ 13
Natterin-3    MKLSVLVVTLLAVSWTSAQPETFSIQ----T-------------------KEANMNPEPA 37
Natterin-4    MKLLVLLVTLLVLSWTSAEDVGDQEILQQHNEDNNHKSELGEAAPQRTDNETSQLGQETP 60
Natterin-1    MIPSVLLVTLLLLSWTSAEKDLK------------------------------------- 23
Natterin-2    MNLSVLLVTLLLLSWTSAEKDLK------------------------------------- 23


CGL1          --------------MAEWVSTTGN-TIPDNAIR--AGYDINKKALFIARAVVSGEMTPGK 43
Natterin-3    NIRVAR--SSSAQSNLQWNYWDGQGAVPDGAVSIWNGEE--KRTDYVCSCGCSSGFYSTK 93
Natterin-4    TIRVARAYEFSSKSNLEWVRWNG--HIPSNAVKISNTYV--GREDYVCRVGCEAGYYTPK 116
Natterin-1    -VRVAR--STNDETNLHWVKCGG--SVPDGAVSIQNTYVSPARTEYVCKSNCEAGYYSTK 78
Natterin-2    -VRVAR--STNDETNLHWVKCGG--SVPDGAVSIRNTYVSPARTEYVCKCFCQAGYYSTK 78
Dln1                             MTYPTNLEIIGGQGGSSFSFTGENNGASLEKIWVWVGGWQI 41

CGL1          CGTHLEGAHIPFAGKEHIIQNYEVLVYPINALGFLDWQQASNGDVPGNAIDTAS--GIYI 101
Natterin-3    T---GANCHYAYGETEKT-CSGFSILVNRDNFENLEWKGGSDGSVPKNAVEVCE--KVYV 147
Natterin-4    K---GPSCFYPYGFTEQH-SKMFHILVNRDNFEILEWKWKTGGEVPENAVKACR--DLYV 170
Natterin-1    D----SKCHYPFGRVEQT-TSVCEILVNRDNFELLEWKEGYAGGSLPANAVSTCKTNRIYV 133
Natterin-2    D----SKCHYPYGTKEMATSTNCYILVNRDNFELLEWKDGYAGGSVPDNAVSTCKTNKIYV 134
Dln1          KAVRAWLSDGRDETFGVPSGSHQEYVFTPGECFTSLSLWGNGAGTRLGAIKFKTNKGGEF 101

CGL1          GRVLYSGSLIPCKIHTGFKVAYMGFAGKEHQSKEYEALYKVI------------------ 143
Natterin-3    GKNKYGL----GKVHTKHEALFLPWHGEEHWYKDYEVLTVNDDVVKQELTQVNYKLDAAH 203
Natterin-4    AKNKYGL----GKLHQSHHVFYLPWKGTEYKYNEYYVLNVNMDVVEQKITNVRYNMKGVE 226
Natterin-1    GKGAYGL----GKIEPAHHCLYYGWNGAETWTKTYQALTVNKDVIEQTMKDVKYQTEGVT 189
Natterin-2    GKGAYGL----GKIEPANHCLYYVWDGAETWTKTYQALTMNKDVIEQAMKDVKYQTEGVT 190
Dln1          FAHMTSWGLKTEYPMDVGSGYCLGIVGRGGSDIDCMGFMFLNAVQSTVLTNVNYPTINQL 161

CGL1          ------------------------------------------------------------
Natterin-3    PIKNPPETLRRSSASNSQCRPITKTVALEKAIQTEQSWDVTSTVTFGVESSITAGIPDIA 263
Natterin-4    VHKDKPETLRSTSVKNYQCREATKQVTLEKSTETSQSWDVSNSITLGVSTEVSAGIPNIA 286
Natterin-1    VIQGKPEVMRKSTVNNKQCKEVTKTVTLSKDISTEERWDVTNSVTFGVTTTVTAGIPDVA 249
Natterin-2    VIKGKPEVMRRSTVNNQHCKEVTKTVTLTKDISTDERWDVTNSVTFGVTTTVTAGIPDVS 250
Dln1          IPKVATEEIKSVSFENKTSVKQEQKVETSKKVIKTSSWSMTKSFSSTFSVEVSAGIPEIA 221

CGL1          ------------------------------------------------------------
Natterin-3    SATVSVSVETSLSVSLGSTTTKTTTHTVSVIVTVPPNHYCPVTMVATKYTADIPFTGKMT 323
Natterin-4    DVSVAVSAETSVEISHGTSKTESTSHSLSVSATIPPNSSCSITMEGCTFKANIPFTGRLT 346
Natterin-1    SASLAVSMEARRDFAHGASKTESQSYMVTVSVPVPPKQSCTVSMVAQVNKADVPFTATLI 309
Natterin-2    SASLEISMQATMDFAHGASKTETQSYMVTVSVPVPPKQSCTVSMVAQVNKADIPFTATLI 310
Dln1          EVSTGFSISFGVESTHSLEQTDEKNETLTTTVEVPPKKKVDVHITIGRASFDLPYTGTVK 281

CGL1          ------------------------------------------------------------
Natterin-3    RTYRNGQKRTTSITGTYRAIQVGEIRADVQRCSEIAGAKPC------------------- 364
Natterin-4    RKYSNGKVTSSSVKGIYKKVQVGEIQAVLHRCDKIADAKPC------------------- 387
Natterin-1    RTYRGGKKTQTTTKGVYRTTQVAETHADVEQCTIIGDEKDCPKASK-------------- 355
Natterin-2    RTYRGGKKTQTTTKGVYRTIQVAETHADVEQCTIIGDAKDCPNASSTITTLRPKLKSKKP 370
Dln1          ITCKNGSVLQYETKGQYKGV----AYTDIKVNTVEKDL---------------------- 315

CGL1          ------
Natterin-3    ------
Natterin-4    ------
Natterin-1    ------
Natterin-2    AKPAGK 376
Dln1          ------
```

Supplementary Figure S4.

Supplementary Figure S4. Alignment of the deduced amino acid sequences of CGL1 and proteins of the Natterin family. The deduced amino acid sequences of CGL1, natterin proteins from *Thalassophryne nattereri* (toadfish), and Dln1 from *Danio rerio* (zebrafish) were aligned in the Clustal Omega software [4]. Residues identical to CGL1 and Dln1 are marked in red and blue, respectively. Residues in the lectin module of Dln1 are marked in yellow. Residues in the signal sequence of natterin proteins are colored green.

```
CGL1_F         1   --MAEWVSTTGNTIPDNAIRAGYDINKKALFIARAVVSGEMTPGKCGTHLEGAHIPFAGKEHIIQNYEVLVYPINA        74
CGL1_L        75   LGFLDWQQASNGDVPGNAIDTA-----SGIYIGRVLYSGSLIPCKIHTGFKVAYMGFAGKEHQSKEYEALYKVI--       143
Q7JZZ3_DROME  74    DHFSWIPSSGGSVPPHAIQVGQTGEGEPLYVGRGYFQGSLTPGKVHPSHQCLYIPYGGQEHRLEAYEVLVQ--PETWIAS 151
D9PTS0_DROME  78    YNYEWLSAENGEVPPGAVKVGRNVDGEYLYAGRGYHAGSLTMGKVHPSHGCLYIPYDSDEVKIFAYEVLCQ--PERWIDT 155
Q8SZ28_DROME  73    QGFAWVPSSSGGVPPNAVRSGTTRTGEPLYVGRGHHAGSLTVGKVHPSHGCLYIPFGGQEVRINTYEVLIKQQHDVWVPA 152
C6SV36_DROME  84    QGFAWVPSSSGGVPPNAVRSGTTRTGEPLYVGRGHHAGSLTVGKVHPSHGCLYIPFGGQEVRINTYEVLIKQQHDVWVPA 163
Q9VBG7_DROME  73    FNYEWLPAENGEVPPGAVKVGQNVDGETLYAGRGYHAGSLTVGKVHPSHGCLYIPYDSEEVKIFAYEVLSR--RLEAR   148
D2NUI1_DROME  81    FNYEWLPAENGEVPPGAVKVGQNVDGETLYAGRGYHAGSLTVGKVHPSHGCLYIPYDSEEVKIFAYEVLSR--RLEAR   156
Q9W2M4_DROME 225    GGGQWLPVDAGNIPPNAVPAGETAEGEPLFIGRATHDGTITVGKVQPSHGCCYIPYGGEELAYKEFEIYV-----      294
Q8MR08_DROME 237    GGGQWLPVDAGNIPPNAVPAGETAEGEPLFIGRATHDGTITVGKVQPSHGCCYIPYGGEELAYKEFEIYV------     306
                        *               *           *                                 *
```

Supplementary Figure S5.

Supplementary Figure S5. Alignment of CGL1 and DM9 repeats in proteins from *Drosophila melanogaster*. Sequences of the first and second half of CGL1 are shown as CGL1_F and CGL1_L, respectively. "######_DROME" means UniProt codes of some proteins from *D. melanogaster*. These sequences from *D. melanogaster* were found to be a DM9 domain and have some homology (34–39%) with CGL1_F. Identical and similar amino acid residues in all of the sequences are shown as red and blue characters, respectively. The amino acid residues identical between the halves of the CGL1 are highlighted in yellow. The residues conserved in more than 80% of proteins bearing the DM9 domain are shown with an asterisk.

A

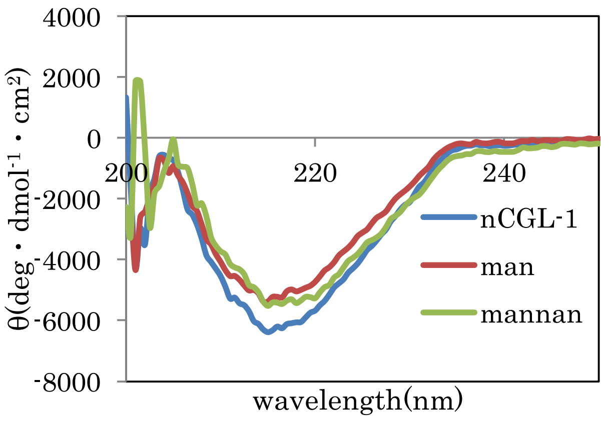

B

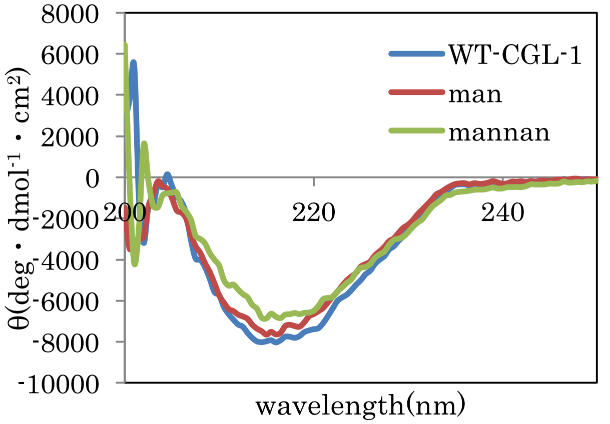

Supplementary Figure S6.

Supplementary Figure S6. Circular dichroism (CD) spectra of native (A) and recombinant (B) CGL1. Far-UV CD spectra of the CGL1 and those in the presence of 10 mM mannose or 10 mg/mL mannan from yeast are blue, red, and green, respectively. The spectra were recorded at 20 °C after dialysis against TBS.

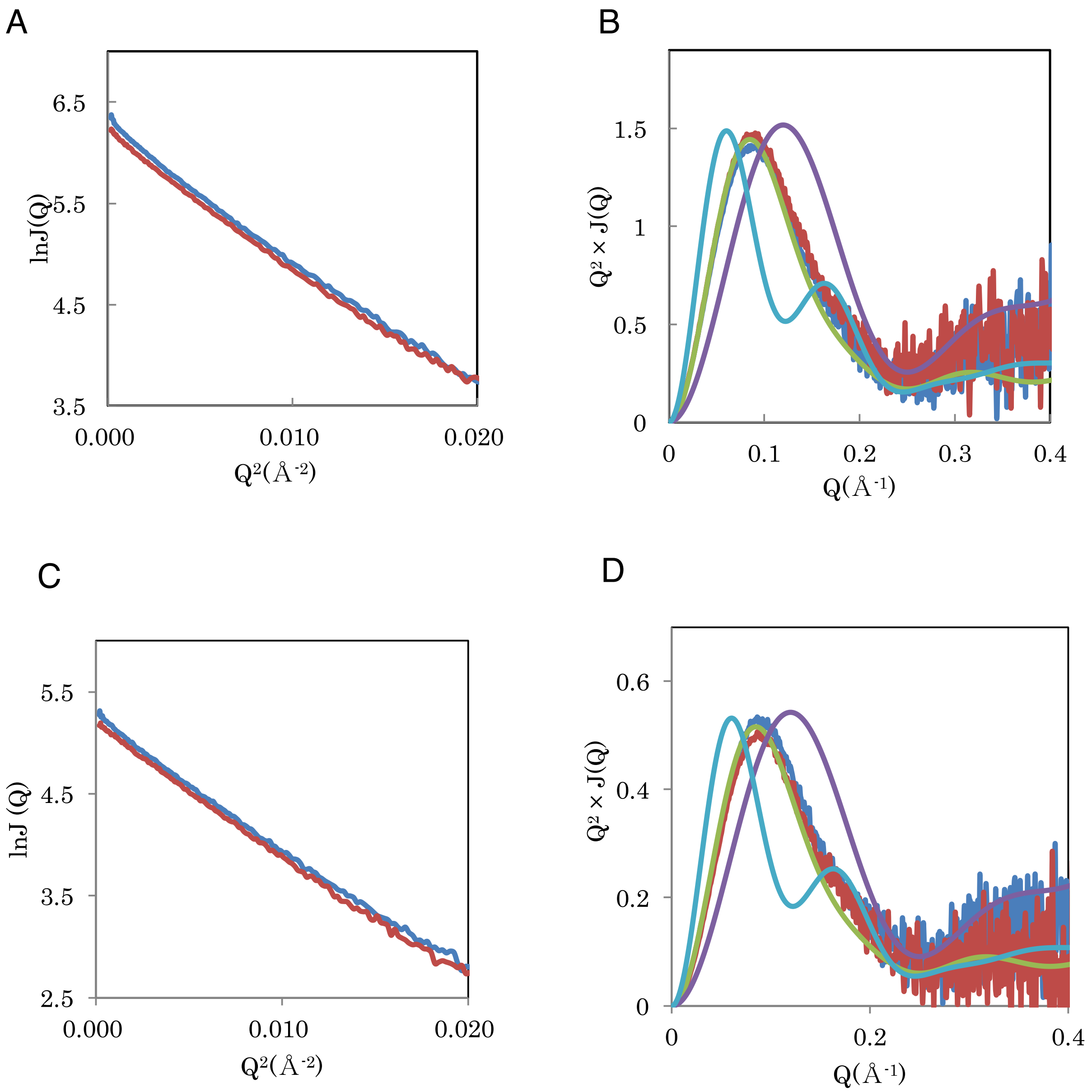

Supplementary Figure S7.

Supplementary Figure S7. Small-angle X-ray scattering of CGL1. The Guinier plot (*A*, *C*) and Kratky plot (*B*, *D*) from the SAXS analyses of the native (*A*, *B*) and recombinant (wild-type) CGL1 (*C*, *D*). The data were obtained in the absence (blue) and in the presence of 100 mM mannose (red). The theoretical curves calculated from the crystal structures of the monomer, dimer, and tetramer (a hypothetical model, 4 protomers are arranged as in the asymmetric unit of CGL1/FREE [purified from *Crassostrea gigas*, dialyzed against TBS, and concentrated to 5 mg/mL] and CGL1/MAN [purified after heterogenous expression in *Escherichia coli*, undialyzed, and concentrated to 10 mg/mL with 100 mM mannose]) are purple, green and light blue, respectively.

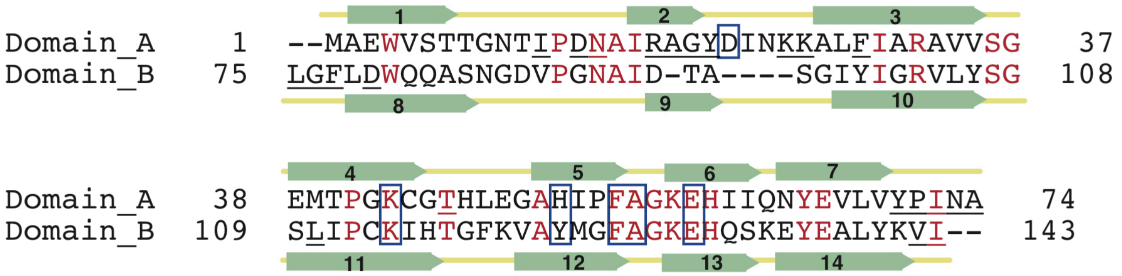

Supplementary Figure S8.

Supplementary Figure S8. Alignment and secondary structure of domains A and B. The arrows represent β-strands. Mannose-binding amino acid residues at sites A and B are in square boxes. The residues responsible for dimerization are underlined. The residues that are conserved between the domains are red.

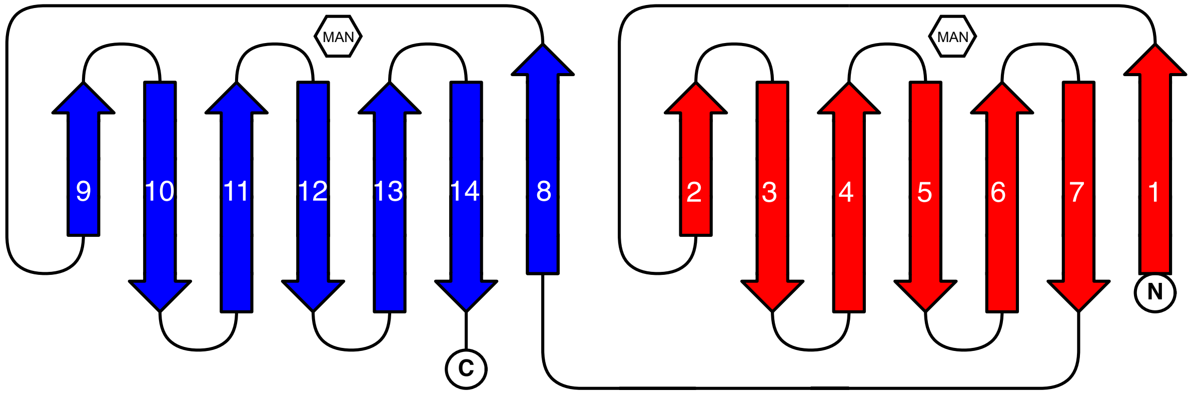

Supplementary Figure S9.

Supplementary Figure S9. A topological diagram illustrating the secondary structural elements of CGL1. Arrows represent β-strands. Domains A and B are red and blue, respectively.

A

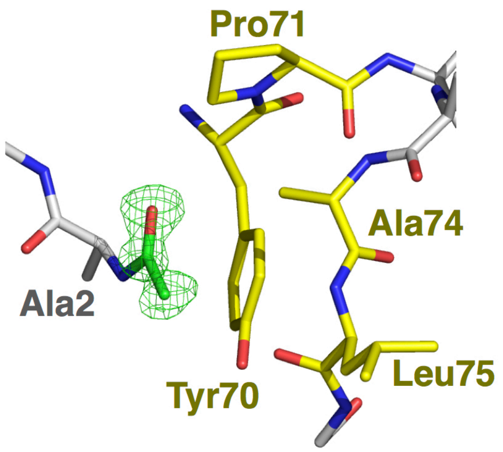

B

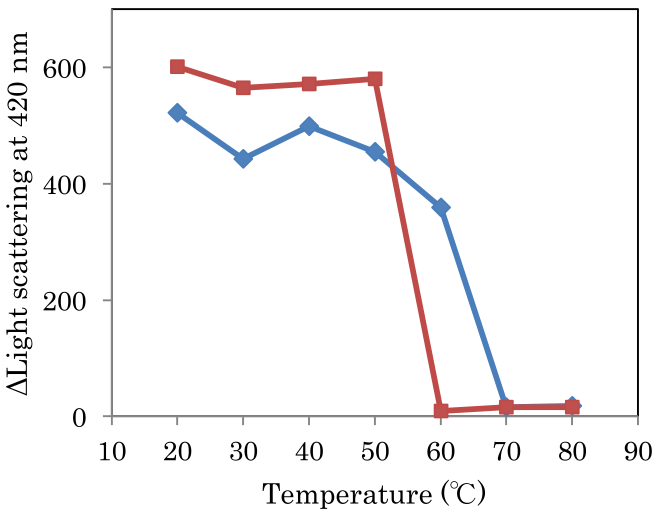

Supplementary Figure S10.

Supplementary Figure S10. *A*, The N-terminal modification of nCGL1. The acetyl group that modifies the N terminus of CGL1 and the interacting amino acid residues are shown as green and yellow stick figures, respectively. The F_o-F_c omit electron density map is shown as a green mesh. The acetyl group was excluded from the calculation of the F_o-F_c omit map. The contour levels of the omit map are at 2.5σ. *B*, The thermal stability of CGL1 according to measurements of light scattering intensity in experiments with a mannotriose-polyamidoamine dendrimer conjugate (mannotriose-PD).-Native (nCGL1) and recombinant (wtCGL1) CGL1s are the blue and red curves, respectively.

REFERENCES

1. Svergun, D., Barberato, C. & Koch, M. H. J. CRYSOL– a Program to Evaluate X-ray Solution Scattering of Biological Macromolecules from Atomic Coordinates. *J Appl Crystallogr* **28,** 768–773 (1995).
2. Hatakeyama, T. *et al.* An assay for carbohydrate-binding activity of lectins using polyamidoamine dendrimer conjugated with carbohydrates. *Biosci. Biotechnol. Biochem.* **76,** 1999–2001 (2012).
3. Laemmli, U. K. Cleavage of structural proteins during the assembly of the head of bacteriophage T4. *Nature* **227,** 680–685 (1970).
4. Sievers, F. *et al.* Fast, scalable generation of high-quality protein multiple sequence alignments using Clustal Omega. *Mol. Syst. Biol.* **7,** 539 (2011).
